# Evaluating the relationship between job stress and job satisfaction among female hospital nurses in Babol: An application of structural equation modeling

**DOI:** 10.15171/hpp.2018.13

**Published:** 2018-04-18

**Authors:** Majid Bagheri Hosseinabadi, Siavash Etemadinezhad, Narges khanjani, Omran Ahmadi, Hemat Gholinia, Mina Galeshi, Seyed Ehsan Samaei

**Affiliations:** ^1^School of Public Health, Shahroud University of Medical Sciences, Shahroud, Iran; ^2^Department of Occupational Health, Mazandaran University of Medical Sciences, Sari, Iran; ^3^Neurology Research Center, Kerman University of Medical Sciences, Kerman, Iran; ^4^Faculty of Medical Sciences, Tarbiat Modares University, Tehran, Iran; ^5^Clinical Research Development Unit of Rouhani Hospital, Babol University of Medical Science, Babol, Iran

**Keywords:** Job Stress, Job Satisfaction, Nurses

## Abstract

**Background:** This study was designed to investigate job satisfaction and its relation to perceived job stress among hospital nurses in Babol County, Iran.

**Methods:** This cross-sectional study was conducted on 406 female nurses in 6 Babol hospitals. Respondents completed the Minnesota Satisfaction Questionnaire (MSQ), the health and safety executive (HSE) indicator tool and a demographic questionnaire. Descriptive, analytical and structural equation modeling (SEM) analyses were carried out applying SPSS v. 22 and AMOS v. 22.

**Results:** The Normed Fit Index (NFI), Non-normed Fit Index (NNFI), Incremental Fit Index (IFI)and Comparative Fit Index (CFI) were greater than 0.9. Also, goodness of fit index (GFI=0.99)and adjusted goodness of fit index (AGFI) were greater than 0.8, and root mean square error of approximation (RMSEA) were 0.04, The model was found to be with an appropriate fit. The R-squared was 0.42 for job satisfaction, and all its dimensions were related to job stress. The dimensions of job stress explained 42% of changes in the variance of job satisfaction. There was a significant relationship between the dimensions of job stress such as demand (β =0.173,CI =0.095 - 0.365, P≤0.001), control (β =0.135, CI =0.062 - 0.404, P =0.008), relationships(β =-0.208, CI =-0.637– -0.209; P≤0.001) and changes (β =0.247, CI =0.360 - 1.026, P≤0.001)with job satisfaction.

**Conclusion:** One of the important interventions to increase job satisfaction among nurses maybe improvement in the workplace. Reducing the level of workload in order to improve job demand and minimizing role conflict through reducing conflicting demands are recommended.

## Introduction

Job stress occurs in the workplace or in relation to job-related factors or due to changes in work activities.^1^ It is an emotional response to the work-related environment and occurs when the conditions and facilities are not suited to the capacities, resources or needs of an employed individual. Personal factors such as shortcomings, characteristics, and methods of dealing with life events are effective on the level of job stress.^2,3^ The adverse effects of job stress are not limited to the work environment and may be seen outside the working environment, as well.^3,4^ According to the American Institute of Stress (AIS), stress is the main cause for about 80% of all job-related injuries and 40% of lacks in workflow within the workplaces.^5^ Huge investments are lost due to staff’s physical and mental diseases, their reduced level of efficiency, job quitting and stress-related job change. Annually, about 1.1 million people lose their lives due to job stress and job-related diseases.^1,6^ Job stress may be happened in all professions, but it seems that nurses face more stress compared to other healthcare staff.^7,8^

Job stress among nurses is a global problem and 9.2% to 68% of nurses may be faced with job stress.^8^ This disorder among nurses may be associated with several psychological (anxiety, depression, exhaustion and poor concentration), physical (increased heart beat rate and blood pressure, cardiovascular diseases and musculoskeletal pains), or organizational (job absenteeism, lack of job satisfaction and lack of quality in job performance) problems.^8^ In addition to job stress, one of the most important organizational issues related to nurses is their level of job satisfaction.^9^

Job satisfaction is a positive and pleasant emotional state and is a result of individual’s assessment on his/her job or job experience.^10^ Job satisfaction may increase level of self-confidence, improve communication, reduce level of psychological distress, and improve physical, mental and social health among nurses.^9^ Job satisfaction among nurses may be also influenced by several factors such as salary, communications, policies, procedures, job dimensions, work order, and personality characteristics.^11,12^

When the needs of nurses are not met at the workplace, they are very likely to experience a variant level of job stress which may negatively impact their job performance and may also lead to job dissatisfaction.^13^ In a study conducted on job satisfaction and its association with staff performance among nurses, there was a positive and significant correlation between job satisfaction and staff performance.^14^ Considering the significant role and the high proportion of female nurses in health care provision, there is a need for better understanding on the psycho-social issues related to their level of job stress and job satisfaction. Better understanding on the relationships between job stress and job satisfaction among female nurses may help healthcare managers, hospital policy makers and nursing instructors in providing appropriate strategies and programs to promote job satisfaction among nurses. Promoting job satisfaction among this population may consequently increase their level of productivity and efficiency, and improve the quality of their performance.^15^

**Figure 1:**
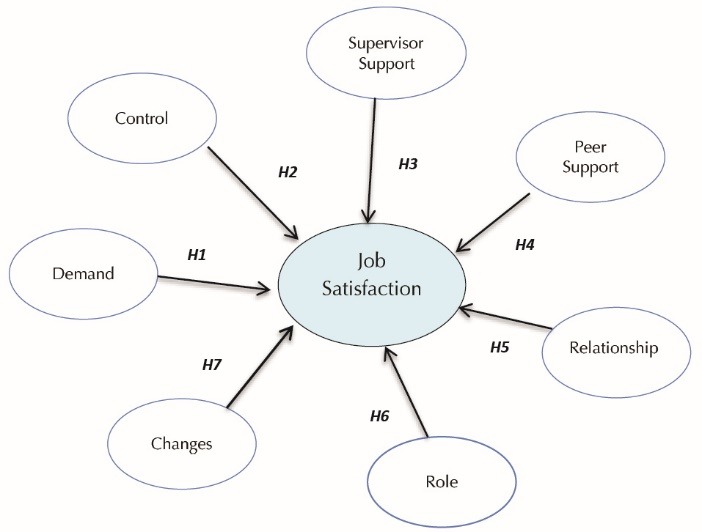
Hypothesized model of the direct effects of perceived job stressors on job satisfaction.

We conducted this study to investigate the level of job satisfaction among nurses and to assess the associations between job stress perceived by nurses in the work environment and their level of job satisfaction. In this study structural equation modeling (SEM) (Figure 1) was used to answer the following question: “may the dimensions of job stress be associated to job satisfaction among female hospital nurses in the governmental hospitals of Babol county, Iran?”

## Materials and Methods

### Study subjects and methods

This cross-sectional study was conducted from April to August 2017. The source population included all female nurses employed in the 6 hospitals affiliated to Babol University of Medical Sciences (N = 1020). In SEM studies, the proportion of 5 to 15 samples per variable is considered to estimate sample size.^16^

**Figure 2:**
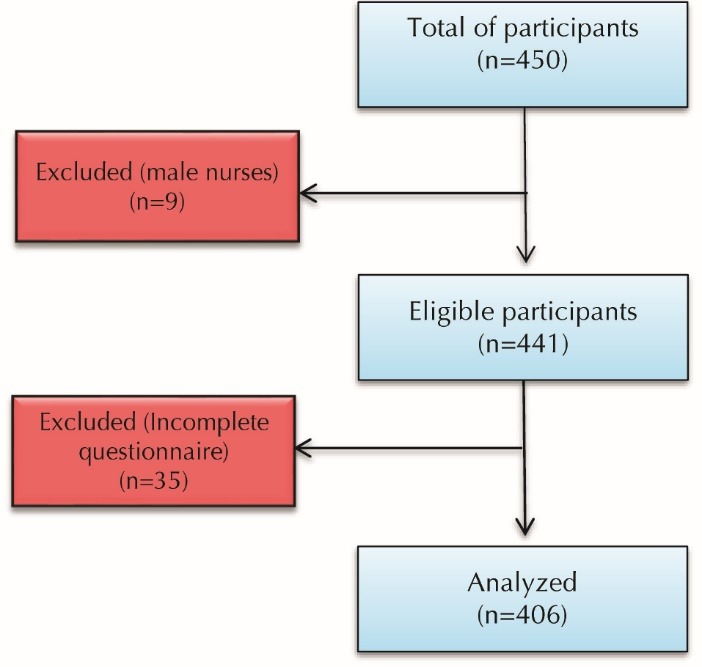
Study flow diagram for participant recruitment.

In our study, there were 55 items in the questionnaire; 35 items for job stress and 20 items for job satisfaction. Thus, 450 nurses were enrolled in the study. Due to the low number of male nurses in the sample (9), we chose to exclude them from analysis. Also, the information of 36 nurses was excluded from data analysis due to incomplete questionnaires. Finally, the data on 406 respondents were analyzed (Figure 2). The respondents were randomly selected based on the table of random numbers. The nurses with at least a bachelor’s degree in nursing, a full-time job, no second job, no specific physical and mental problems (based on self-report) and at least one year of work experience in the current employment were included in the study. The exclusion criteria included not willing to participate in the study and not complete the whole questionnaire.

### Measures

In this study the following questionnaires were used for data collection.

a) Demographic and organizational information checklist which was a researcher-made form including questions on age, section or workplace, total work experience (years), and level of education.

b) HSE Indicator Tool: This standard job stress questionnaire was developed by the UK Health and Safety Executive (HSE) team, which includes 35 items with 7 subscales: 1. Demand (includes topics such as workload, characteristics, and work environment), 2. Control ( the extent to which a person is on the path to perform her duties), 3. Officials’ support (the amount of support that a person receives from the management and social service), 4. Colleagues’ support (the amount of support that a person receives from her own colleagues), 5. Relationships (practice and positive features to increase inter-personal communication and reduce conflict in the workplace), 6. Role (staff’s correct understanding about their job role in the organization), 7. Changes (how to organize for change factors in an organization). The response format is based on a 5-point Likert-type scaling (never, rarely, sometimes, often and always). The Cronbach α for this scale were reported to be 0.78 and 0.81 in the studies done by Mahdavi et al^17^ and Marzabadi and Gholami Fesharaki,^18^ respectively. Internal consistency for the dimensions of questionnaire in this study was estimated to be 0.6-0.75 (Table 1).

c) The Minnesota Satisfaction Questionnaire (MSQ): this questionnaire was used to measure job satisfaction. MSQ is one of the widely used tools to measure job satisfaction in different jobs. This questionnaire includes 20 items with two subscales: intrinsic and extrinsic job satisfaction. Intrinsic satisfaction is referred to the working conditions and the individual’s feeling about the nature of his job and duties, and extrinsic satisfaction is related to the environmental conditions and the individual’s feeling about characteristics of the job out of his working environment. The participants should express their satisfaction with their current job by responding through a 5-point Likert-type scale (from “highly dissatisfied” to “highly satisfied”). The MSQ-short form has been widely used in Iranian settings and has been shown to have a good reliability and validity.^19,20^ The total satisfaction score ranges from 20 (low job satisfaction) to 100 (high job satisfaction). In this study the Cronbach’s alphas for intrinsic, extrinsic and total satisfaction were 0.85, 0.71 and 0.88, respectively (Table 1).

### Statistical analysis

Descriptive statistics was used to summarize the data. SEM was used to investigate the relationships between latent and observed variables. SPSS v. 22 and Amos v. 22 were applied to analyze data. The significance level was considered to be *P *< 0.05, a priori.

### The details of SEM model specification

At first, the model was developed based on literature review. Then, the methods of measuring variables such as reflection and combination of hidden variables and relationships between them were determined.

*Model identification:* The number of data in the model was more than unknown models. As a result, there were unique answers for the models that were unknown.

*Model estimation:* The maximum likelihood method was used to estimate the parameters of the model. It was assumed that the data was normal.

*Model testing:* In assessment process of the model, the variance of the proposed model with standard covariance was compared with fitness indices.

*Model modification:* In order to improve the model, if needed variables were deleted or covariances were added between the variables and errors.

*Model validation:* The validation of model was evaluated by fitness indicators and recommended amounts.

## Results

More than half of the respondents were at the range of 30-39 years of age. About 20% were working at the general wards and 66.3% had less than 10 years of work experience. Only 8.2% had a master or PhD degree in nursing (Table 2).

Among the dimension of the job stress, the highest score was for role dimension (mean ± standard deviation: 4.28 ± 0.57) (Table 1).

### Correlation between variables

Based on Pearson correlation coefficient test, there were significant correlations between the dimensions of job stress and job satisfaction (Table 3).

### Structural equation modeling results

**Figure 3:**
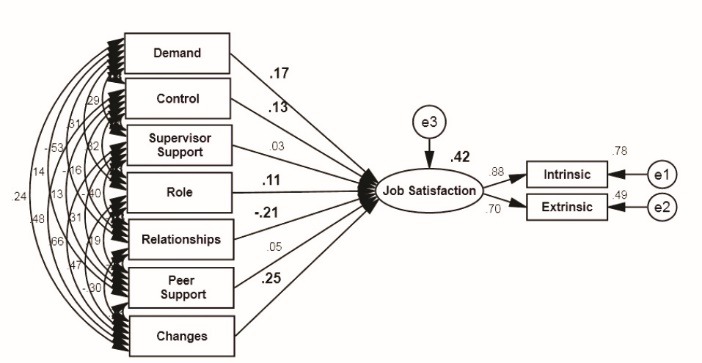
Model in the mode of standard coefficients.

The recommended model (Figure 1) was determined in Amos software v. 22 and the path analysis results are shown in Figure 3. In the figures, the final research model in standard and significant modes is provided which shows the general model in the standard estimation mode. In this case, there is a possibility to compare between the observed variables that explain the hidden variables. This standardized model indicates the amount of variance related to hidden variables explained by the observed variables.

### The fitness of the proposed model

Our results showed χ2 to be equal to 10.55 with degree of freedom (*df*) = 6 (*P* = 0.000); GFI = 0.99; AGFI = 0.96; RMSEA = 0.04; NFI = 0.99; CFI = 0.99. These findings suggested that the hypothesized model fitted the data well and the model had an appropriate fit (Table 4).

### Result of structural equation modeling 

In this study, several hypotheses were examined based on the standard coefficients and t values. Based on this approach, the hypothesis was accepted, because the t-value was not at the range of -1.96 to +1.96.

There were significant relationships between the dimensions of demand, control, relationships and changes with job satisfaction. The R-squared values was estimated 0.42 for job satisfaction which showed all dimensions of job stress in our study in association with job satisfaction. These dimensions explained 42% of the total changes in the independent variable (Table 5).

## Discussion

Job stress may affect the mental health of staff and consequently reduce the level of job satisfaction.^21^ Marcatto et al^22^ in 2014 investigated the relationships between stress, satisfaction and job content among 760 Italian nurses. They used a similar questionnaire and found the highest mean scores for role and colleagues’ support subscales and the lowest mean score for the changes subscale among the dimensions of job stress. The final stress score in their studywas less than those found in the present study. In another study conducted by Edwards et al^23^ among 30 903 employees throughout the United Kingdom similar findings were reported. The final stress score in their study^23^ was found to be 3.53 ± 0.03 which was similar to that found in our study. In a study among British police officers, the highest and lowest mean were for demand and changes subscales, respectively.^24^ These findings suggest that the role subscale is the most influential factor in determining job stress and may cause job burnout and dissatisfaction. Nurses have multiple roles in the healthcare system. Usually, they do not have a clear description of their job which may result in a lack of role transparency among them.^25^ In nursing, each role has its own specific characteristics and the expectations for a role may be in conflict with the demand for another role. Therefore, role conflict may occur.^26^

According to our results, the level of internal, external and total job satisfaction among the nurses were 2.83 ± 0.61, 3.02 ± 0.68, and 2.89 ± 0.57, respectively. A study conducted among 524 Chinese nurses showed the level of job satisfaction to be 2.51 ± 0.98^27^ which was less than those found in our study. However, the nurses who participated in our study reported lower level of job satisfaction compared to the Ethiopian and Jordanian nurses.^28,29^

In SEM, we found significant relationships between job satisfaction with demand, control, role, and changes. Moreover, there was a negative significant relationship between job satisfaction and relationships. Marcatto et al also reported significant relations between job satisfaction with demand, control, supervisor’ support, relationships, and role.^22^ The model fit in their study was 0.42. The participants included in the study of Marcatto and colleagues^22^ were the staff of legal offices, local police, the education sector, social services, the cultural sports section and marketers who were different from the participants of our study. Therefore, the difference in the nature of jobs may be the cause of some differences in the results of the studies.

In our study, there was a direct relation between job satisfaction and demands and a reverse relation between job satisfaction and relationships. The dimensions of demand and poor relationships in the environment are important factors in increasing the level of job stress. In numerous previous studies, demand (workload) and job efforts have been shown as critical factors in increasing the level of job stress.^30,31^ The effect of poor relationships in the workplace on job stress is also logical and may affect the perceived stress in the workplace. However, relationships may change over time and vary from person to person or from workplace to another.

In our study, there was a significant direct relation between job satisfaction and role. Ho et al, in a study conducted on 532 nurses in 2 Thai hospitals showed role conflict as the most important cause of job dissatisfaction among the nurses.^32^ Previous studies have also shown nursing as a job associated with multiple and contradictory demands from the supervisors, managers, doctors and the executive staff.^33,34^ Such a situation may result in role conflict and may deteriorate when nurses are faced with acute and deadly diseases.^35^

The results of this study showed a direct relation between job control and job satisfaction. Athey et al investigated the relation between job control (independence in decision-making) and job satisfaction among 8311 nurses in the US hospitals. Their study showed job control as the most important predictor for job satisfaction.^36^ Also, Iliopoulou and While in their study on 431 special care nurses showed a positive and significant relation between job control and job satisfaction and no significant relation between job satisfaction and job conflict and ambiguity.^37^ Job control provides nurses with authority to make decisions about patient care. Therefore, job satisfaction among nurses may be promoted through improvement in independence for decision making.^38^

According to our results, changes were another domain of job stress that associated to job satisfaction. This domain deals with investigating how to manage changes at work. Kerr et al showed significant association between job satisfaction and changes.^39^ The results of our study and those of other studies indicate that high level of changes in an organization may cause more job insecurity feelings among the nurses and this can have a negative effect on the individuals’ job satisfaction.^40,41^ Therefore, reducing the level of changes and subsequently reducing job insecurity may be effective in improving job satisfaction among nurses.

## Limitations

A limitation in our study was the use of self-reported questionnaires to collect data. The psycho-emotional situation of an individual when completing a questionnaire may affect the answers. In order to reduce this effect, the nurses were asked to select an appropriate time to complete the questionnaire. Another limitation was the cross-sectional nature of the study. Stress and job satisfaction may change over time due to changes in the workplace. Also, all the study population were female. The number of male nurses was low and we chose not to recruit male nurses for our study. Considering gender differences in understanding job satisfaction and stress, further studies should be conducted on both genders. As another limitation, work shift in this study were classified into rotating shifts and fixed shifts, and therefore the association between type of work shift (morning, evening, and night) and job dissatisfaction may not be understood. Finally, job satisfaction is a multifactorial issue which may be affected by several factors besides stress. It is recommended for future studies on the determinants of job satisfaction to consider a various range of factors such as job burnout, the duration of work experience, the shift-work, income of participants and job demand.

## Conclusion

In our study, job satisfaction was directly associated to demand, control, relationships, role, and changes and was reversely associated to relationships. Interventional efforts are suggested to increase job satisfaction among nurses through improving the workplace situation and reducing workload with the hope to improve job demand, decisions making, applying therapeutic methods, improving relationships skills, and minimizing role conflict among nurses.

## Ethical approval

The researchers visited the hospitals after obtaining permission from Babol University of Medical Sciences (ethical No. MUBABOL.HRI. REC 1395.254). Nurses were provided with the questionnaires after informing them about the goals of the study individually and obtaining their consent. Data were recorded anonymously. The researcher was available to answer the participants’ questions.

## Competing interests

There are no conflicts of interest.

## Authors’ contributions

MBH and SES and SA involved in the conception and designing the study. HG and NK performed the data analysis and interpretation. SES wrote the manuscript and acted as corresponding author. OA and MG supervised the development of work, helped in data interpretation and manuscript evaluation. NK helped to evaluate and edit the manuscript.

## Funding

The authors received no financial support for the research, authorship, and/or publication of this article.

## Acknowledgments

The authors thank the nurses and authorities Babol University of Medical Sciences hospitals for their cooperation and Clinical Research and Development Unit of Ayatollah Rouhani Hospital in Babol, Iran.

**Table 1:** Mean and standard deviation of HSE and MSQ items reported by nurses (n = 325)

	**Item**	**Mean**	**SD**	**Min**	**Max**	**Skewness**	**Cronbach’s alpha**
HSE	Demand	2.88	0.64	1	4.75	0.015	0.75
	Control	3.14	0.64	1.5	5	-0.054	0.6
	Supervisor Support	3.64	0.71	1.6	5	-0.107	0.7
	Peer Support	3.70	0.69	1	5	-0.461	0.74
	Relationships	2.45	0.83	1	5	0.365	0.72
	Role	4.28	0.57	1.8	5	-0.918	0.71
	Changes	3.41	0.78	1.33	5	0.128	0.61
	Total	3.64	0.38	2.5	4.88	0.195	0.77
MSQ	Intrinsic Satisfaction	2.83	0.61	1.31	5	-0.045	0.85
	Extrinsic Satisfaction	3.02	0.68	1	5	-0.291	0.71
	Total	2.89	0.57	1.21	5	-0.05	0.88

Abbreviations: HSE, Job Stress Questionnaire; MSQ, Minnesota Satisfaction Questionnaire.

**Table 2:** Individual and organizational characteristics of the participating nurses

**Variables**	**Category**	**Total**
Age (y)	20-29	92 (22.6)
	30-39	238 (58.6)
	≥40	76 (18.8)
Ward	Emergency ward	21 (5.2)
	Critical care unit	84 (20.7)
	General ward	80 (19.7)
	Surgery wards	146 (36)
	Operating room	75 (18.5)
Clinical experience (y)	≤10	269 (66.3)
	11-20	118 (29.1)
	>20	19 (4.7)
Educational level	BSN^a^	373 (91.8)
	MSN/PhD^b^	33 (8.2)

^a^ Bachelor of Science in Nursing. ^b^ Master of Science in Nursing/ Doctor of Philosophy in Nursing.

**Table 3:** Correlation between job stress and job satisfaction among nurses

**Variables**	**Changes**	**Peer Support**	**Relationships**	**Role**	**Supervisor Support**	**Control**	**Demand**	**Satisfaction**
Changes	1.000							
Peer Support	0.226**	1.000						
Relationships	-0.301**	-0.213**	1.000					
Role	0.465**	0.190**	-0.428**	1.000				
Supervisor Support	0.656**	0.308**	-0.396**	0.627**	1.000			
Control	0.479**	0.133**	-0.160**	0.325**	0.375**	1.000		
Demand	0.238**	0.136**	-0.525**	0.313**	0.294**	0.276**	1.000	
Satisfaction	0.451**	0.205**	-0.416**	0.382**	0.411**	0.343**	0.378**	1.000

**Correlation is significant at the 0.01 level (2-tailed).

**Table 4:** Examining fitness indicators

**Fitting indexes**	**Full name**	**Recommended amount**	**Value**	Confirm/Reject
χ^2^	Chi-square divided	-	10.55	Confirm
*df*	Degrees of freedom	-	6	Confirm
χ^2^/*df*	Chi-square divided to degrees of freedom	χ2/df < 3	1.75	Confirm
RMSEA	Root mean square error of approximation	RMSEA ≤ 0.10	0.04	Confirm
NNFI	Non-normed fit index	NNFI > 0.9	0.97	Confirm
NFI	Normed fit index	NFI > 0.9	0.99	Confirm
AGFI	Adjusted goodness of fit index	AGFI > 0.9	0.96	Confirm
GFI	Goodness of fit index	GFI > 0.9	0.99	Confirm
CFI	Comparative fit index	CFI > 0.9	0.99	Confirm
IFI	Incremental fit index	IFI > 0.9	0.99	Confirm

**Table 5:** Estimates for the structural parameters in Figure 1

**Model**		**Standardized coefficients (β)**	**95% CI**	***t*** ** value**	***P*** ** value**	**Hypothesis**
H1	Demand → Satisfaction	0.173	(0.095,0.365)	3.308	<0.001	Accepted
H2	Control → Satisfaction	0.135	(0.062,0.404)	2.667	0.008	Accepted
H3	Supervisor Support → Satisfaction	0.030	(-0.191,0.303)	0.445	0.657	Reject
H4	Peer Support → Satisfaction	0.054	(-0.082,0.338)	1.198	0.231	Reject
H5	Relationships → Satisfaction	-0.208	(-0.637,-0.209)	-.820	<0.001	Accepted
H6	Role → Satisfaction	0.106	(-0.023,0.533)	1.804	0.071	Reject
H7	Changes → Satisfaction	0.247	(0.360,1.026)	4.071	<0.001	Accepted

R-squared = 42.0.
